# Scent of a Killer: Microbial Volatilome and Its Role in the Biological Control of Plant Pathogens

**DOI:** 10.3389/fmicb.2020.00041

**Published:** 2020-02-07

**Authors:** Bruno Tilocca, Aocheng Cao, Quirico Migheli

**Affiliations:** ^1^Department of Health Sciences, University “Magna Græcia” of Catanzaro, Catanzaro, Italy; ^2^Dipartimento di Agraria and NRD-Nucleo di Ricerca sulla Desertificazione, Università degli Studi di Sassari, Sassari, Italy; ^3^Institute of Plant Protection, Chinese Academy of Agricultural Sciences, Beijing, China

**Keywords:** volatile organic metabolites, biocontrol, antagonist, yeast, eco-friendly agriculture

## Abstract

The use of synthetic fungicides represents the most common strategy to control plant pathogens. Excessive and/or long-term distribution of chemicals is responsible for increased levels of environmental pollution, as well as adverse health consequence to humans and animals. These issues are deeply influencing public perception, as reflected by the increasing demand for safer and eco-friendly agricultural commodities and their by-products. A steadily increasing number of research efforts is now devoted to explore the use of safer and innovative approaches to control plant pathogens. The use of microorganisms as biological control agents (BCAs) represents one of the most durable and promising strategies. Among the panoply of microbial mechanisms exerted by BCAs, the production of volatile organic compounds (VOCs) represents an intriguing issue, mostly exploitable in circumstances where a direct contact between the pathogen and its antagonist is not practicable. VOCs are potentially produced by all living microorganisms, and may be active in the biocontrol of phytopathogenic oomycetes, fungi, and bacteria by means of antimicrobial activity and/or other cross-talk interactions. Their biological effects, the reduced residuals in the environment and on agricultural commodities, and the ease of application in different agricultural systems make the use of VOCs a promising and sustainable approach to replace synthetic fungicides in the control of plant pathogens. In this review, we focus on VOCs produced by bacteria and fungi and on their role in the cross-talk existing between the plant pathogens and their host. Biologic systemic effect of the microbial volatile blends on both pathogen and host plant cells is also briefly reviewed.

## Introduction

Synthetic biocides are the major route to control plant pathogens (Irtwange, 2006). However, it has been widely demonstrated that prolonged usage of such agrochemicals is associated with unsustainable levels of environmental pollution, hence raising ecological concern. Long-term exposure to synthetic fungicides recorded a reduced treatment efficacy due to the development of resistance mechanisms by plant pathogens. This led farmers to increase chemical application, with a consequent accumulation of residues in the agricultural commodities and their by-products which, in turn, are responsible for harmful effects for both human and animal health (Pal and McSpadden Gardener, 2006). In addition, the appearance of resistance mechanisms represents a clinical problem, and resistance to fungicides among human pathogenic fungi is being increasingly observed (Cowen et al., 2002; Mehta et al., 2018). A typical example is represented by the acquired resistance of *Aspergillus fumigatus* and other human pathogenic fungi to azoles (i.e., one of the major class of fungicides, widely used in both agricultural and clinical treatment) as reviewed by Deising et al. (2008).

These reasons have a strong influence on the public perception and market demand, posing the need to move toward the production of pesticide-free commodities, in a healthier and more ecologically friendly context. In this view, a promising pest management alternative is represented by the biological control approach, where the human intervention exploits the natural antagonistic effects of some agents (i.e., the biological control agents, or BCAs) to mitigate the detrimental effects of pathogenic (micro)organisms (Irtwange, 2006). Biological control mechanisms exerted by BCAs are diverse and depend on the specific peculiarities of both pathogen and the antagonist, as well as their density and the specificity of the interactions occurring among these species (Pal and McSpadden Gardener, 2006). A successful BCA is generally featured by the activation of a plurality of mechanisms and targets, synergistically aimed at controlling the pathogen and/or its detrimental effect (Droby et al., 2009). In addition, ideal BCAs do not produce toxic metabolites for both humans/animals and the environment (D’Alessandro et al., 2014; Velivelli et al., 2015). Direct antagonism (e.g., hyperparasitism and predation) occurs in the case of a very high affinity among the pathogen and its BCA (Wisniewski et al., 1991; Heydari and Pessarakli, 2010). Here, interacting species get directly in physical contact and the BCA exerts its suppressive effect without the need for any auxiliary activity from other microorganisms or the surrounding environment (Pal and McSpadden Gardener, 2006). Contrariwise, in the case of indirect antagonism (e.g., competition, or host resistance induction), no physical contact is required between the BCA and its target (Heydari and Pessarakli, 2010). Instead, the BCA acts as a “stimulus” to trigger the development of an unfavorable condition for the microbial growth, leading to a control of the pathogenic species (Pal and McSpadden Gardener, 2006).

Other pathogen suppression mechanisms include the production of volatiles, antibiotics, and other secondary metabolites of the microbial lifecycle. Volatile organic compounds (VOCs) production is gaining a constantly increasing interest by the scientific community, owing to the diverse advantages of their application. VOCs are a blend of volatile metabolites potentially produced by all living microorganisms and were observed to be active in the control of phytopathogenic oomycetes, fungi, and bacteria by means of antimicrobial activity and other cross-talk interactions. Their antimicrobial effects, along with the reduced hazard for both environment and human beings and their possible application without the need of a supplemental spray or drench, make the use of VOCs a promising and sustainable approach to replace fungicides of synthetic origin in the control of plant pathogens (Mercier and Jiménez, 2004; Fialho et al., 2010; Parafati et al., 2017).

In the current review, we focus our attention on the VOCs production by BCAs, intended as active effectors of the dynamic network of cross-relations existing among microbial entities and their host. Their potential exploitation as effective mechanisms to control the causal agents of diseases of economically relevant plants is discussed.

## Volatile Organic Compounds of Microbial Origin

The volatile metabolites of both microbial and plant origin are gaining a steadily increasing interest, and the term “volatilome” has been relatively recently adopted to refer to this complex heterogeneous ensemble of metabolites (Maffei et al., 2011; Farbo et al., 2018).

Among the volatile metabolites produced by the microbial and/or plant metabolism, organic and inorganic molecules can be primarily differentiated. Inorganic volatile molecules such as CO, CO_2_, H_2_, N_2,_ O_2_, NH_3_, H_2_S, NO_2_^-^, SO_2_, SO_3_, and HCN are the most relevant and are involved in a wide variety of biological functions ranging from electron acceptors/donor to acting as defense compound (Effmert et al., 2012). Moreover, a role in the interspecies communication (e.g., quorum sensing/quenching) and antibiotic resistance has been recently proved (Schmidt et al., 2015; Avalos et al., 2019). By acknowledging the important role of inorganic volatile metabolites under diverse biological and ecological aspects, the current review deals mainly with volatile organic compounds of microbial origin and their role in biological control of plant pathogens.

**Figure 1:**
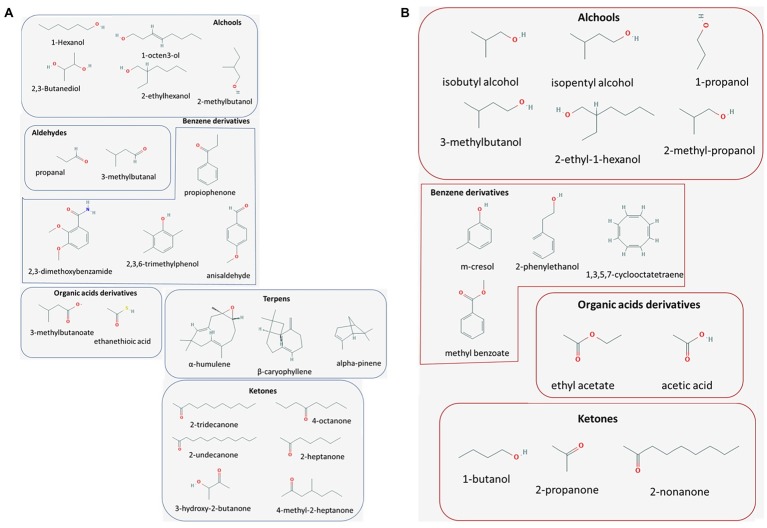
Major volatile metabolites with antimicrobial properties. Molecular classes of bacterial (blue framed, panel **A**) and fungal (red framed, panel **B**) origin reported to exert promising antimicrobial activities, of actual or potential application in the biological control of plant pathogens.

Volatile organic compounds are small (typically less than 300 Da), carbon-based molecules featured by a low water solubility and a high vapor pressure that makes them available in a gaseous status in the normal ambient conditions (i.e., 1 atm pressure and 25°C temperature) (Pagans et al., 2006; Vespermann et al., 2007; Morath et al., 2012). From a chemical point of view, VOCs are comprised of a heterogeneity of molecular classes, including hydrocarbons, alcohols, thioalcohols, aldehydes, ketones, thioesters, cyclohexanes, heterocyclic compounds, phenols, and benzene derivatives (Wheatley et al., 1997; Chiron and Micherlot, 2005; Morath et al., 2012). Examples of the most commonly investigated volatile molecules of bacterial and fungal origin are provided in Figures 1A,B, respectively.

In agriculture, the use of VOCs of microbial origin in the biocontrol of plant pathogens has received a reduced marginal in the past years; however, progresses recently made, along with the overall tendency of the scientific community toward the adoption of a holistic approach, highlighted the potential benefits of microbial VOCs in this field.

VOCs are generally effective already at a very low concentration (Mitchell et al., 2010; Raza et al., 2016). Being volatile, VOCs are capable of diffusing between the soil particles and spread in the atmosphere over large distances from their application point, where they can exert their inhibitory activity without requiring a direct or physical contact between the VOCs-producing microorganism and the target pathogen (Minerdi et al., 2009; Heydari and Pessarakli, 2010). Besides pathogen inhibition and the negative effects on fungal spore germination and the activity of morphogenesis enzymes (Fialho et al., 2011), microbial VOCs have also shown to be involved in a wide variety of processes (McKee and Robinson, 2009; Morath et al., 2012; Yuan et al., 2012). These include the capability of VOCs to kill plant-parasitic nematodes (Gu et al., 2007; Yang et al., 2012; Xu et al., 2015), the ability to promote plant growth (Ryu et al., 2003; Mercier and Manker, 2005; Minerdi et al., 2011), and the induction of resistance mechanisms in plants, preventing them from being colonized by pathogens (van Loon et al., 1998; Compant et al., 2005; Farag et al., 2006).

Biosynthetic pathways leading to the production of microbial VOCs are yet poorly understood; however, omics-based studies so far performed link the VOCs production to either metabolic transformation products of lipids, proteins, and other building blocks of living tissues, or as the result of degradation (i.e., end-products) of catabolic reactions (Serrano and Gallego, 2006; Tholl et al., 2006; van Dam and Poppy, 2008; Kännaste et al., 2014). On this basis, a dichotomic classification of VOCs as primary or secondary metabolites appears rather inappropriate; instead, VOCs are commonly classified on the basis of their molecular features, such as the number of carbon atoms, ring moieties, and substituent groups (Bennett et al., 2012).

The composition of microbial VOC blends depends on several factors. These include the microbial entities (e.g., bacteria, fungi) producing the VOCs, the substrate they are grown on, temperature, radiation, presence of other microorganisms, and the type of ecosystem (Pasanen et al., 1997; Nilsson et al., 2004; Fiedler et al., 2005). Also, VOCs composition of a given species is highly dynamic over time, resulting in a changing composition of the produced VOCs depending on the age of the VOCs-producing species (Wang et al., 2013).

Regardless of the emitting species and the specific VOCs blend composition, several studies are nowadays being performed with the aim to exploit the countless benefits of employing a natural antimicrobial mixture to prevent plant pathogens by replacing traditional chemical approaches. Unanimously, studies performed so far recommend VOCs application under air-tight environment, in order to rapidly saturate the atmosphere with the volatile antimicrobials. Particularly effective application of VOCs is reported in the control of storage pathogens on fresh fruit (e.g., citrus, peach, strawberry) but also other commodities such as nuts, grains, and seeds (Table 1; Strobel, 2006; Fialho et al., 2011; Wang et al., 2013; Chen et al., 2018; Gao et al., 2018).

**Table 1:** Main biological control agents emitting volatile organic compounds, their target pathogen, framework application, and primary volatilome components.

Antagonist	Target	Application	Main VOCs	Reference
*Aureobasidium pullulans*	*Botrytis cinerea* *Colletotrichum acutatum* *Penicillium expansum* *Penicillium digitatumPenicillium italicum*	Post-harvest decay toxin contamination	2-Phenylethanol	Di Francesco et al. (2015)
*Cyberlindnera jadinii* *Lachancea thermotolerans* *Candida intermedia* *Candida friedrichii*	*Aspergillus carbonarius* *Aspergillus ochraceus*	Post-harvest decay toxin contamination	2-Phenylethanol	Farbo et al. (2018) Tilocca et al. (2019)
*Saccharomyces cerevisiae*	*Phyllosticta citricarpa*	Post-harvest decay	2-Phenylethanol	Fialho et al. (2010)
*Wickerhamomyces anomalus*	*Aspergillus flavus*	Toxin contamination	2-Phenylethanol	Hua et al. (2014)
*Hanseniaspora uvarum* *Pichia kluyveri* *Wickerhamomyces anomalus*	*Aspergillus ochraceus*	Toxin contamination	2-Phenylethyl acetate	Masoud et al. (2005)
*Aureobasidium pullulans* *Metschnikowia pulcherrima* *Saccharomyces cerevisiae* *Wickerhamomyces anomalus*	*Botrytis cinerea*	Pathogen prevention/inhibition	Whole volatilome	Parafati et al. (2015)
*Candida sake*	*Botrytis cinerea* *Penicillum expansum*	Post-harvest decay	Whole volatilome	Arrarte et al. (2017)
*Bacillus amyloliquefaciens*	*Fusarium oxysporum*	Pathogen prevention	2,3,6-Trimethyl-phenol Pentadecane Tetradecane	Yuan et al. (2012)
*Bacillus atrophaeus*	*Botrytis cinerea*	Pathogen prevention/inhibition	Hexadecane 2,3-Dimethoxybenzamide Oanisaldehyde	Zhang et al. (2013)
*Burkholderia ambifaria*	*Rhizoctonia solani* *Alternaria alternata*	Pathogen prevention/inhibition	Dimethyldisulfide 2-Undecanone dimethyltrisulfide 4-Octanone Methylmethanethiosulfonate Phenylpropanone	Groenhagen et al. (2013)
*Burkholderia tropica*	*Colletotrichum gloeosporioides* *Fusarium culmorum* *Fusarium oxysporum* *Athelia rolfsii*	Pathogen prevention/inhibition	Limonene Alpha-pinene Ocimene	Tenorio-Salgado et al. (2013)
*Burkholderia gladioli*	*Fusarium oxysporum* *Rhizoctonia solani*	Pathogen prevention/inhibition	Limonene	Elshafie et al. (2012)
*Achromobacter* sp. *Serratia* sp.	*Fusarium oxysporum*	Plant growth induction	Dimethyldisulfide Propanal 2-Ethyl-1-hexanol Dodecane Tridecane Tetradecane	Minerdi et al. (2009, 2011)
*Bacillus* spp.	Host	Resistance induction Plant growth induction	3-Hydroxy-2-butanone 2,3-Butanediol	Ryu et al. (2003) Rudrappa et al. (2010)
*Enterobacter aerogenes*	*Exserohilum turcicum*	Pathogen prevention/inhibition	2,3-Butanediol	D’Alessandro et al. (2014)
*Phomopsis* sp.	*Pythium* spp. *Phytophthora* spp. *Sclerotinia* spp. *Rhizoctonia* spp. *Fusarium* spp. *Botrytis* spp. *Verticillium* spp. *Colletotrichum* spp.	Pathogen prevention/inhibition	Sabinene 1-Butanol 3-Methylbenzeneethanol 1-Propanol 2-Methyl 2-Propanone	Zhou et al. (2014)
*Phaeosphaeria nodorum*	*Monilinia fructicola*	Pathogen prevention/inhibition	3-Methylbutanol Acetic acid 2-Propyl-1-ol Ethyl acetate	Naznin et al. (2012, 2014)
*Wickerhamomyces anomalus*	*Penicillium roqueforti*	Post-harvest decay	Ethyl acetate	Liu et al. (2014)
*Trichoderma viride*	*Arabidopsis thaliana*	Plant growth induction	Isobutyl alcohol Isopentyl alcohol 3-Methylbutanal	Hung et al. (2013)
*Talaromyces* sp.	*Colletotrichum higginsianum*	Plant growth induction	β-Caryophyllene	Di Francesco et al. (2015)
*Cladosporium* sp. *Ampelomyces* sp.	*Pseudomonas syringae*	Resistance induction pathogen Prevention/inhibition	m-Cresol Methylbenzoate	Di Francesco et al. (2015)

### Bacterial Volatilome as a Tool for the Biocontrol of Plant Pathogens

Bacteria produce volatile metabolites as part of their normal metabolism (Figure 1A). Bacterial VOCs are involved in the complex network of interconnections established among bacterial species, bacteria vs. other microorganisms, and bacteria vs. plants. Such interactions have a variable ecological role, ranging from beneficial cooperation (e.g., mutualism, symbiosis, host resistance induction) to antagonistic relationship occurring, for instance, in the case of microbicidal activity exerted by one of the interacting species (Maffei et al., 2011; Kanchiswamy et al., 2015). The recent awareness on the beneficial effects arising from bacteria-plant interaction opens new avenues in the use of bacterial volatilome to stimulate plant growth. Moreover, owing to the high versatility of bacteria-derived VOCs and their effectiveness in controlling other microorganisms, studies are focusing on exploiting the natural bacterial VOCs production as a strategy for the biocontrol of plant pathogens.

In this view, only a handful of studies have so far been performed to elucidate the metabolic effects of the bacterial volatilome on the target organism, while it is well known that bacteria-derived VOCs have a pivotal role in stimulating or repressing other bacterial species (Garbeva et al., 2014a; Kanchiswamy et al., 2015).

*Bacillus amyloliquefaciens* strain SQR-9 has been reported as effective against the tomato wilt pathogen *Ralstonia solanacearum* (Raza et al., 2016). A key role is played by the VOCs blend produced by the BCA, which provides effective inhibition of *R. solanacearum* in both synthetic media and in soil. Inhibitory effects of the bacterial VOCs have been confirmed by lack of inhibition observed in the case of treatment with a non-VOCs-producing bacterium as well as when *B. amyloliquefaciens* SQR-9 is applied in the presence of activated charcoal (i.e., a well-known gas adsorbent). Growth inhibition was dependent on the BCA load; however, the inhibitory effects produced by the bacterial VOCs were reversed by BCA removal, indicating a bacteriostatic effect of the *B. amyloliquefaciens-*derived VOCs on *R. solanacearum* (Raza et al., 2016). Bacteriostatic evidences were also observed in the volatilome of different strains of *Pseudomonas chlororaphis, Serratia plymuthica IC1270,* and *Serratia proteamaculans* 94, tested for their antagonistic potential against *Agrobacterium tumefaciens* C58 (Popova et al., 2014). In dual culture, all strains succeeded in total or partial inhibition of the phytopathogenic bacterium. An exception is represented by *S. proteamaculans* 94, resulting in a non-significant inhibition of the pathogen. Nevertheless, this strain scored a total inhibition of the cyanobacterium *Synechococcus* sp. PCC 7942 and other eukaryotic cells (Popova et al., 2014). This study confirms previous reports on the *in vitro* antagonistic potential of *S. plymuthica* IC1270, *P. fluorescens* Q8r1-96, and *P. fluorescens* B-4117 against phytopathogenic *A. tumefaciens* and *Agrobacterium vitis*. Here, authors indicated VOCs produced by the candidate BCAs as a valuable tool to prevent crown gall tumors on tomato plants (Dandurishvili et al., 2011).

Investigations on the composition and activity of the bacterial VOCs blend revealed a strain-specific VOCs mixture, with some molecular entities being exclusive to a given bacterial strain, whereas quantitative changes were observed among the other “commonly identified” volatile molecules. This might justify the diverse antagonistic features of candidate BCAs in regard to different pathogens (Dandurishvili et al., 2011; Popova et al., 2014; Garbeva et al., 2014b). Dimethyl disulfide (DMDS) was identified as the major volatile produced by *S. proteamaculans* 94 and other *Serratia* spp. (Dandurishvili et al., 2011; Popova et al., 2014); however, only traces of DMDS have been produced by *Pseudomonas* spp. strains, in favor of the production of ketones. Among these compounds, 2-nonanone, 2-heptanone, 2-undecanone, and 2-tridecanone are among the most represented bactericidal compounds as confirmed in subsequent VOCs experiments (Dandurishvili et al., 2011; Popova et al., 2014; Raza et al., 2016).

Bacteria-bacteria interaction does not necessarily result in the sole bactericidal (or bacteriostatic) effect; it may also imply a synergistic/cooperative activity among bacterial species. A recent investigation performed on soil bacteria showed that the volatilome of four genetically diverse isolates results in antithetical phenotypes of *P. fluorescens*. Here, VOCs released by *Collimonas pratensis* and *S. plymuthica* positively stimulated the growth of *P. fluorescens*, with *C. pratensis* volatiles even stimulating the production of antimicrobial compounds by *P. fluorescens*. On the other hand, *P. fluorescens* exposure to *Paenibacillus* and *Pedobacter* spp. did not stimulate the growth of *P. fluorescens*, but triggered a stress response mechanism in the bacterial model even though no significant inhibition was observed for any of the four different strains (Garbeva et al., 2014b).

Besides intra-kingdom interconnections, bacteria are also involved in sophisticated bi-directional cross-talks involving phylogenetically higher species such as fungi and plants: bacteria-fungi interactions are very dynamic, depending on the interacting species and are strongly exploited in the modern agricultural practice to control important phytopathogenic taxa (Maffei et al., 2011).

Rhizobacteria such as *S. plymuthica*, *Serratia odorifera, Stenotrophomonas maltophilia*, *Stenotrophomonas rhizophila, P. fluorescens,* and *Pseudomonas trivialis* are known to produce VOCs mixtures with antifungal properties active against a wide array of both pathogenic and non-pathogenic fungi (Kai et al., 2010; Effmert et al., 2012; Kanchiswamy et al., 2015). Common volatile molecules known in the bacteria-fungi interaction are γ-patchoulene, 3-methylbutanal, 1-octen3-ol, 2-undecanone, 2-nonanone, 3-methylbutanoate, 2-methylbutan-1-ol, 4-methyl-2-heptanone, ethanethioic acid, and dimethyltrisulfide 2,3,6-trimethylphenol. Among these, several have already been tested for their antifungal activity.

A study on the antifungal activity of *B. amyloliquefaciens* NJN-6 volatilome demonstrated the ability of this bacterium to hinder growth and spore germination of the pathogenic *Fusarium oxysporum* f. sp. *cubense* causing fusarium wilt on banana. Analysis of its volatilome composition identified a total of 36 volatile molecules, including aromatic compounds, alkyls, ketones, alcohols, naphthyls, aldehydes, one ester and one ether compound. *In vitro* evaluation of the identified compounds suggested an important fungicidal activity of benzothiazoles phenol and 2,3,6-trimethylphenol. Other benzenic compounds, instead, were attributed to the “sole” inhibitory properties, owing to their inability to preclude the growth of the pathogenic fungus. The other identified compounds were able to inhibit almost completely *F. oxysporum* growth only when present in massive quantities, leading authors to exclude them as potential candidates to antagonize *F. oxysporum* growth (Yuan et al., 2012).

Volatiles released by *Streptomyces* spp. have also shown interesting antifungal properties, especially in the control of storage and mycotoxigenic fungi (Schöller et al., 2002; Li et al., 2010). A further study by Wang and colleagues confirmed the simultaneous antifungal activity of *Streptomyces alboflavus* TD-1 with regard to some of the most known mycotoxigenic fungi such as *F. moniliforme* (*syn. Fusarium fujikuroi*)*, A. flavus, Aspergillus ochraceus, Aspergillus niger,* and *Penicillum citrinum* (Wang et al., 2013). Moreover, the investigation of its volatilome underlines a very complex and dynamic composition, with several molecules shared among taxonomically related species even though with high quantitative variability (Wilkins and Schöller, 2009; Li et al., 2010; Wang et al., 2013). In addition, some volatiles have been previously identified as components of essential oils of diverse plants with already supposed antifungal effects (Bajpai et al., 2008; Essien et al., 2011; Serrano et al., 2011; Shanthi et al., 2011; Wang et al., 2013; Gao et al., 2018). The mix of volatiles produced by *Bacillus atrophaeus* CAB-1 strains is mainly composed of hexadecane, 2,3-dimethoxybenzamide and oanisaldehyde. *In vitro* assay of the bacterial volatiles resulted in an effective inhibition of *Botrytis cinerea*, the causal agent of tomato gray mold (Zhang et al., 2013). In another study, DMDS, 2-undecanone, dimethyltrisulfide (DMTS), 4-octanone, S-methylmethanethiosulfonate, and 1-phenylpropan-1-one produced by *Burkholderia ambifaria* scored a significant inhibition of the growth of the pathogenic fungi *Rhizoctonia solani* and *Alternaria alternata* (Groenhagen et al., 2013). Similarly, the investigation of 15 strains of *Burkholderia tropica* resulted in a significant inhibition of the growth of four fungal pathogens, namely *Colletotrichum gloeosporioides, Fusarium culmorum, F. oxysporum*, and *Athelia rolfsii*, most likely due to their capability to produce VOCs, among which limonene, alpha-pinene, dimethyldisulfide (DMDS), and ocimene were considered as the most effective ones (Tenorio-Salgado et al., 2013). The antifungal activity of limonene was also confirmed in a further study, where the growth rate of *F. oxysporum* and *R. solani* has been hindered by the limonene emitted by *Bulkholderia gladioli* pv. *agaricola* (Elshafie et al., 2012).

Besides antagonism, VOCs from several bacterial species are also involved in ectosymbiotic relations occurring between bacteria and fungi. The bacterial volatiles of *Achromobacter* and *Serratia* spp. DMDS, propanal, 2-ethyl-1-hexanol, dodecane, tridecane, and tetradecane, are capable of stimulating *F*. *oxysporum* MSA35 to produce a higher amount of α-humulene and β-caryophyllene which, in turn, are known to favor lettuce growth (Minerdi et al., 2009, 2011).

### Bacterial Volatilome Prevents Pathogen Infection by Conditioning Plant Physiology

Interactions occurring between plants and bacteria, by means of volatile emission, may have both beneficial and detrimental outcomes for the overall plant growth (Kai et al., 2009; Gutiérrez-Luna et al., 2010). Elucidating the complex cross-talk occurring between bacteria and their host allows a conscious human intervention that promotes plant growth by preventing plant colonization and/or by exploiting the direct beneficial effects that some bacteria exert on plants. Blom and co-workers screened over 40 bacterial species for their capability to stimulate plant growth. The study selected 36 bacterial volatiles and, of these, indole, 1-hexanol and pentadecene showed the most promising results in terms of plant growth stimulation (Blom et al., 2011). On the other hand, co-cultivation of *A. thaliana* in the presence of *Serratia odorifera* volatilome resulted in a severe inhibition of plant growth. Authors attributed the inhibitory effect to DMDS and ammonia volatiles (Vespermann et al., 2007; Kai et al., 2010). With regard to DMDS and its direct (i.e., plant growth stimulation) and indirect (e.g., disease prevention) effects on plants, controversial results are available in the literature. A study performed on *Nicotiana attenuata* reports DMDS as a volatile metabolite that directly alters plant metabolism, causing the downregulation of sulfur assimilation and methionine biosynthesis genes (Meldau et al., 2013). Contrariwise, a previous study linked the bacterial production of DMDS to a significantly increased growth of *A. thaliana* (Groenhagen et al., 2013). Accordingly, another study reported that DMDS produced by *Bacillus cereus* results in significant protection of tobacco and corn from the infection by *B. cinerea* and *Bipolaris maydis*, respectively (Huang et al., 2012a).

Other bacterial metabolites involved in bacteria-plant cross-talk include 3-hydroxy-2-butanone and 2,3-butanediol. These are produced by *Bacillus* spp. and are linked to a significantly enhanced total leaf surface area besides the induction of host systemic resistance (Ryu et al., 2003; Rudrappa et al., 2010). This observation is also confirmed in another study highlighting 2,3-butanediol produced by *Enterobacter aerogenes* as involved in the induction of resistance against *Exserohilum turcicum* infection in corn plants (D’Alessandro et al., 2014).

### Fungal Volatilome and Its Role in Biological Control

As for bacteria, several fungal species produce volatile metabolites (Figure 1B) taking part in diverse ecological relationships (Kanchiswamy et al., 2015), ranging from antagonism to symbiotic relations with other fungi (Schiestl et al., 2006), bacteria, and plants (Hung et al., 2013).

Among antagonistic fungi, *Muscodor albus*, an endophyte from *Cinnamomum zeylanicum* (cinnamon tree), represents the first commercially available BCA acting through its volatilome (Strobel et al., 2001; Strobel, 2006). This fungus produces a wide variety of volatiles comprising alcohol, acid, ester, and terpenoid derivatives, with antimicrobial properties against pathogens responsible of the post-harvest decay of fruits such as apple, peach, lemon, and grape. Moreover, it proved safe for humans and the environment, enabling its registration as a biopesticide at the US Environmental Protection Agency. The usage of bags containing a lyophilized culture of *M. albus* that is reactivated by hydration is advantageous for the biofumigation under air-tight environments, providing an effective prevention of fruit decay during storage and shipping (Strobel, 2006; Mercier et al., 2010). Moreover, due to its remarkable antagonistic activity, the use of *M. albus* was already proposed by Strobel and colleagues as a valuable alternative to replace methyl bromide fumigation, with comparable results (Strobel, 2006; Strobel et al., 2011). A risk assessment for the effects of VOCs on human health and the environment did not show important harmful potential since these compounds are produced in low amounts, do not contaminate treated food commodities, and dissipate rapidly in the atmosphere. Nevertheless, it has been recently demonstrated that a volatile molecule produced by *M. albus* raised human health issues, hence underlying the need to develop further studies/guidelines for a comprehensive assessment of the potential toxicity exerted by VOCs produced by BCAs (Braun et al., 2012; Jiménez et al., 2012; Romanazzi et al., 2012; Margolis, 2012, personal communication).

Other endophytic fungi have shown to promote plant growth through the release of volatile metabolites inhibiting the growth of plant pathogens. Investigations on endophytes from *Rosa x damascena* (Damask rose) yielded over 50 isolates, among which *A. niger* was found to produce high amounts of 2-phenylethanol (2-PE; Wani et al., 2010). In addition to the potential value in the cosmetic industry, this study is among the first ones linking the production of 2-PE with its potential application as fumigant, owing to its antimicrobial and antiseptic properties already exploited in pharmaceutics (Wani et al., 2010). Other studies indicated 2-PE as the major component of yeast volatile blends and described its role in limiting the growth of pathogenic *Aspergillus* spp. (Fialho et al., 2010; Hua et al., 2014; Liu et al., 2014; Chang et al., 2015; Farbo et al., 2018). *Phomopsis* spp. isolated by *Odontoglossum* sp. (*Orchidaceae*) emits a mixture of volatile metabolites including sabinene, 1-butanol, 3-methyl, benzene ethanol, 1-propanol, 2-methyl, and 2-propanone. An artificial mixture of such volatiles had remarkable antifungal properties against several oomycete and fungal pathogens, including *Pythium*, *Phytophthora*, *Sclerotinia*, *Rhizoctonia*, *Fusarium*, *Botrytis*, *Verticillium*, and *Colletotrichum* spp. In addition, the fungus was shown to tolerate and survive in the presence of the volatile metabolites produced by the antagonistic *M. albus* (Mercier and Jiménez, 2004; Singh et al., 2011). *Epichloe typhina* isolated from *Phleum pratense* emits sesquiterpene volatiles with antifungal activity against *Cladosporium phlei* (Kumar and Kaushik, 2012). *Phaeosphaeria nodorum*, a common endophyte of *Prunus domestica*, produces a blend of volatile metabolites comprising ethyl acetate, 3-methylbutan-1-ol, acetic acid, 2-propyl-1-ol, and 2-propenenitril. Altogether, these volatiles inhibit the pathogenic fungus *Monilinia fructicola*, by reducing its growth, hyphal width, and by provoking the disintegration of the hyphal content (Pimenta et al., 2012).

### Yeast Volatilome and Its Effects Against Pathogenic and Mycotoxin-Producing Fungi

The application of yeasts as BCAs represents one of the most investigated alternatives to fungicides, due to the great ability of these microorganisms to grow and survive in heterogeneous ecological niches and under severe stress conditions (Muccilli and Restuccia, 2015). Moreover, the highly competitive activity of yeasts does not suffer the side effects (e.g., production of human allergenic compounds or toxic secondary metabolites) sometimes encountered with the application of other microbial species as BCAs, therefore extending their potential application to the eco-friendly safeguard of agricultural commodities and by-products (Droby et al., 2009; Janisiewicz et al., 2010; Liu et al., 2013; Muccilli and Restuccia, 2015). Early studies performed on *Wickerhamomyces anomalus* (syn. *Pichia anomala*) have shown that the antimicrobial effectiveness of yeast-derived volatilome is mainly attributed to ethyl acetate, enabling for a successful control of *Penicillium roqueforti* during air-tight storage of grain (Ädel Druvefors and Schnürer, 2005). Nevertheless, recent evidence confirmed the impossibility to determine a static inhibitory mechanism. As for bacteria, the yeast-pathogen interaction, as well as the chemical composition of the emitted blend of volatiles, is widely dynamic in relation to several factors such as the VOCs-producing yeast, the antagonized pathogen and the ecological niche where the cross-talking species are growing (Mari et al., 2012; Yuan et al., 2012; Parafati et al., 2017). Volatile metabolites produced by *Sporidiobolus pararoseus* effectively inhibited spore germination and mycelial growth of *B. cinerea*. Investigation of the volatile blend composition highlighted 2-ethyl-1-hexanol as the major compound (Huang et al., 2012b); whereas 1,3,5,7-cyclo octatetraene, 3-methyl-1-butanol, 2-nonanone, and phenylethyl alcohol are the major components of the VOCs produced by *Candida intermedia* antagonizing the same pathogen (*B. cinerea*) both *in vitro* and *in planta* (Huang et al., 2011).

*Meyerozyma guilliermondii* has shown antifungal activity against the rice blast pathogen *Pyricularia oryzae* by means of VOCs production, with ethyl-acetate (Coda et al., 2013) and helvolic acid (Zhao et al., 2010) being the most effective molecules. More recent studies performed on *W. anomalus* identified 2-PE as the most effective compound in preventing spore germination, mycelial growth, and toxin production by *A. flavus* (Hua et al., 2014). Fiori et al. (2014) highlighted the effect of two non-fermenting (*Cyberlindnera jadinii* and *Candida friedrichii*) and of two low-fermenting (*C. intermedia* and *Lachancea thermotolerans*) yeast strains, resulting in the inhibition of both mycelial growth and toxigenic potential of the pathogen *Aspergillus carbonarius*. Subsequently, the chemical composition of the four yeast strains volatilome was characterized: although more than 20 different compounds were identified as components of the yeast-derived volatilome, 2-PE was found to be the most abundant one in all tested volatile blends (Farbo et al., 2018). A recent proteomic investigation aimed at assessing the role of *C. intermedia* volatilome in the inhibition of the ochratoxin A (OTA) producing fungus *A. carbonarius* revealed that yeast VOCs target a plurality of fungal metabolic routes, inducing a marked reduction of the protein biosynthetic activity, proliferative activity, energy metabolism, and inhibiting the fungal detoxification system. Nevertheless, exposure to the sole 2-PE (i.e., the major volatilome component) can only partially reproduce the metabolic alteration provoked by the whole yeast-derived volatilome, thereby suggesting that other minor and still unidentified yeast VOCs components are likely to involve a plurality of metabolic targets, resulting in a higher effectiveness of the treatment over the long-term period (Tilocca et al., 2019). In line with these observations, a very recent study reported the higher antagonistic efficiency of bacterial and fungal volatilome considered “as whole” when compared with the administration of the blend components in their pure form (Mülner et al., 2019).

Volatile organic compounds produced by *W. anomalus, Pichia kluyveri,* and *Hanseniaspora uvarum* inhibited the mycotoxigenic fungus *A. ochraceus* growth as well as OTA production during processing of coffee (Masoud et al., 2005; Masoud and Kaltoft, 2006).

Volatiles produced by *Aureobasidium pullulans* have been tested against *B. cinerea*, *Colletotrichum acutatum, P. expansum, P. digitatum*, and *P. italicum* resulting in an effective control of these post-harvest fruit pathogens growth both *in vitro* and *in planta* (Di Francesco et al., 2015). In a similar study, Parafati and colleagues attributed to VOCs produced by *W. anomalus*, *Metschnikowia pulcherrima*, *S. cerevisiae*, and *A. pullulans* a pivotal role in the biocontrol of *B. cinerea* vegetative growth and its infection rate on table grape berries (Parafati et al., 2015). Similarly, volatiles produced by *Candida sake* reduced the incidence of apple rot caused by the storage pathogens *P. expansum* and *B. cinerea* (Arrarte et al., 2017).

### Exploiting Fungus-Plant Cross-Talk to Control Pathogens

Besides the pathogen-antagonist relationship, plant-fungi interactions are also widely exploited because of the beneficial effects exerted either directly or indirectly on the plant growth.

Direct effects of fungi on plant growth promotion are mainly investigated for endophytes, a group of plant-associated fungal symbionts emitting a wealth of volatile molecules with heterogenous physical, chemical, and biological properties (Khan et al., 2014; Waqas et al., 2014; Zhou et al., 2014; Kanchiswamy et al., 2015). Previous investigations showed that tobacco seedlings are improved by *Phoma* sp. GS8-3 volatiles, with a wide array of C4–C8 hydrocarbons being produced by this endophytic strain (Naznin et al., 2012). Although VOCs blend composition appeared highly dynamic over fungal growth, 2-methyl-propanol and 3-methyl-butanol were the most abundant compounds along the whole fungal cultivation period (Naznin et al., 2012). Volatile metabolites from endophytic fungi have demonstrated to induce plant growth when administered both as singular molecules and as a whole blend of compounds (Khan et al., 2014; Waqas et al., 2014; Zhou et al., 2014; Kanchiswamy et al., 2015). Quite remarkably, the application of low concentration of the volatiles blend has registered a better plant growth promotion than that observed upon exposure to higher concentrations (Naznin et al., 2012, 2014).

A total of 51 diverse volatiles have been identified from the fungal volatilome of *Trichoderma viride*, the most abundant of which included isobutyl alcohol, isopentyl alcohol, and 3-methylbutanal. Dual culture of *T. viride* with *A. thaliana* under air-tight condition revealed a significant plant growth promotion, stimulating bigger, taller, and earlier flowering plants (Hung et al., 2013).

Indirect effects of fungi on the plant growth concern primarily the induction of systemic resistance, providing the plants with the adequate resources to face infection by pathogenic species. The plant-growth promotion exerted by the fungi *Cladosporium* and *Ampelomyces* spp. can be partly attributed to their volatilome, with m-cresol and methyl benzoate, respectively, being the major players in eliciting host systemic resistance, hence resulting in a significantly decreased disease severity after experimental infection with *Pseudomonas syringae* pv. *tomato* DC3000 (Naznin et al., 2014). Another study, aiming at characterizing the volatilome of *Talaromyces* spp., identified several terpenoid-like molecules including β-caryophyllene. Subsequent investigation of the sole β-caryophyllene on *Brassica campestris* L. var. *perviridis* resulted in a significantly increased growth of seedlings and enhanced resistance against *Colletotrichum higginsianum* infection (Yamagiwa et al., 2011).

### Volatile Organic Compounds Production by Mixed Microbial Consortia

It has been already proven that the heterogeneous ensemble of VOCs produced by a single organism may result in different biological outcomes (Tilocca et al., 2019). Analogously, multiple microbial specimens coexisting in the same ecological niche (e.g., the soil) might lead to differing biocontrol achievements compared to what observed and/or expected by the application of a single microbial entity. Many types of interaction can occur among microbial strains, genera, phyla, and even kingdoms (Delory et al., 2016; Schulz-Bohm et al., 2017), leading to a diverse overall behavior of the microbiota, intended as a whole unique entity, whose biological and ecological role is the result of all interactions occurring among all microbiota members. It has been recently proved that interactions occurring among the microbiota members *Bacillus cereus Rs-MS53* and *Pseudomonas helmanticensis Sc-B94* result in enhanced effectiveness while controlling the pathogenic fungus *R. solani* (Mülner et al., 2019), supporting previous evidences of a strong strain compatibility and cooperative interaction (Lim et al., 1991; Dowling and O’Gara, 1994; Ait-Lahsen et al., 2001; Hong and Meng, 2003; Peighami-Ashnaei et al., 2009; Asari et al., 2016). Several strains of *Pseudomonas* and *Bacillus* spp. have shown to produce both volatile and nonvolatile antimicrobial compounds, resulting in either a direct inhibition of the pathogen or a conditioning of the whole microbial community that hampers pathogens growth and infection (Schulz-Bohm et al., 2017). Analogously, it has been reported that *Collimonas pratensis* and *Serratia plymuthica* inhibit the growth of pathogenic *Bacillus* sp. by means of an indirect stimulation of *Pseudomonas fluorescens* and its subsequent stimulation of antimicrobial compounds production (Garbeva et al., 2014b). Microbial cross-talk plays also a pivotal role in the microbial persistence, including antimicrobial resistance and optimal exploitation of scarce nutritional resources (Raza et al., 2016; Jones et al., 2017).

Microbiota investigation is still in its infancy under all applicative fields and further investigations are certainly needed. The soil-associated microbiota is rather complex, mostly because of the high biological diversity comprised in its architecture and the myriad of interfering molecules sampled along that hamper a fair and accurate analysis. Nevertheless, technical progress in the field of *meta-omics* sciences amended disciplines such as metagenomics, metatranscriptomics metaproteomics, and metabolomics, enabling a comprehensive investigation of the taxonomical composition, metabolic potential, and effective metabolic function of all microbial members. Such holistic approach would greatly benefit the comprehensive understanding of the whole microbial community and how it can be shaped by acting on key variables (e.g., physicochemical characteristics of the soil, inclusion of “probiotics”). Moreover, by analogy with animal-oriented research (Tröscher-Mußotter et al., 2019), investigating how the soil/plant microbiota interacts with its plant host, system biology would provide precious information to be exploited in diverse applicative field, including biological control.

## Practical Application of Microbial Volatile Organic Compounds

Volatile blends emitted by BCAs have resulted in a high effectiveness even at low concentrations. Moreover, the reduced release of residual and the negligible hazardous effects on both animals and the environment makes BCA volatilome an intriguing alternative to the use of synthetic pesticides and/or fertilizers. In addition, the high volatility of these molecules enables a wide and homogeneous diffusion both below- and above-ground level. On the other hand, volatility of these natural metabolites is also responsible for the major challenge to their massive application in open-field agricultural and horticultural practices. Drenching of 2,3-butanediol, 3-pentanol, and 2-butanone revealed reproducible outcomes (Cortes-Barco et al., 2010a,b). Moreover, open field application of the 3-pentanol and 2-butanone in a cucumber field has demonstrated a significant effectiveness against the bacterial angular leaf spot pathogen *Pseudomonas syringae* pv*. lachrymans* by inducing plant systemic acquired resistance mechanisms. In turn, the activation of the defense-related gene CsLOX stimulated the oxylipin pathway, which plays a role in recruiting *Coccinella septempunctata*, a natural enemy of the sucking insect aphid, *Myzus persicae* (Song and Ryu, 2013).

Similar observations were reported by a field study performed on red pepper (Choi et al., 2014). Treatment with *Bacillus amyloliquefaciens* strain IN937a on plant leaves resulted in an antagonistic effect against *Xanthomonas axonopodis* pv. *vesicatoria*. The 3-pentanol of bacterial origin has proved to be effective in the induction of plant resistance mechanism by priming salicylic acid, jasmonic acid, and ethylene defense signaling pathway. Another soundly piece of evidence supporting microbial VOCs in the open-field practices has been reported by D’Alessandro and colleagues, who demonstrated that field application on maize plants of acetoin and 2,3-butanediol produced by *Enterococcusaerogenes* triggers a higher resistance against the Northern corn leaf blight fungus *Setosphaeria turcica,* most likely by stimulating the plant defense system (D’Alessandro et al., 2014).

In a recent investigation performed on potato field, five bacterial strains, namely *Pseudomonas palleroniana* R43631, *Bacillus* sp. R47065, R47131, *Paenibacillus* sp. B3a R49541, and *Bacillus simplex* M3-4 R49538, were designed as suitable to improve potato yield by means of VOC production (Velivelli et al., 2015). Nevertheless, molecular details on how the single components of the volatilome exert their inhibitory activity are generally missing in field studies. On the other hand, studies carried out *in vitro* and/or greenhouse condition do generally succeed in the evaluation of the biological mechanisms triggered by the microbial VOCs, but lack to consider the practicability under open-field conditions. Undoubtedly, investigation of the microbial VOCs is still in its infancy and further complementary studies are needed to design appropriate methods for delivery and effective lasting of these innovative treatments (Velivelli et al., 2015; Chung et al., 2016; Schulz-Bohm et al., 2017).

To date, the most promising results were achieved by applying microbial VOCs for the control and prevention of storage pathogens. VOCs application under air-tight condition ensures a rapid saturation of the atmosphere and allows the maintaining of concentration levels of the microbial volatile blend above the minimal effective concentration required to succeed with the pathogen control strategies. In this view, application of microbial VOCs in close environment condition may represent a valuable approach for investigating unique molecules and complex VOCs mixtures in relation to their potential biocontrol activity. Identification of bioactive molecules along with the dynamic cross-talk these compounds are involved in would greatly facilitate the development of suitable chemical forms (e.g., immobilized molecules, pro-bioactive compounds) that should allow better handling, storage, and safe deliver to open fields (Kanchiswamy et al., 2015).

## Conclusion

Microbes produce a wide array of volatile metabolites linked to a complex network of interactions, involving intra- and inter-species relationship. Most of the studies so far performed have focused on the unidirectional effect of the microbial VOCs produced by a single microbial entity in regard to another microbial species or strain (Schulz-Bohm et al., 2015, 2017; Splivallo et al., 2015). Although of a great importance, this “foreshortening of the microbial reality” is rather simplistic and does not consider a wealth of biotic and abiotic factors that would facilitate a comprehensive understanding of the whole ecosystem the microbial species are coping with; thus, how modulating the ecosystem can shape the overall VOCs composition, in favor of a better control of the pathogen diffusion and plant growth stimulation.

Moreover, we are now suffering from a lack of knowledge related to VOCs emission by protists, archaea, or other rhizosphere organisms, such as nematodes or earthworms (Schulz-Bohm et al., 2017). These groups are currently understudied with respect to this aspect, yet their contribution to the overall VOCs composition and the inter-species cross-talk may be of crucial importance. In this view, we foresee that effort to validate this promising strategy will focus on expanding the knowledge on the microbial VOCs biodiversity along with the investigation of their effect on the living community through the adoption of holistic approaches such as -omics sciences and bioinformatics prediction tools.

## Author Contributions

All authors listed have made a substantial, direct and intellectual contribution to the work, and approved it for publication.

### Conflict of Interest

The authors declare that the research was conducted in the absence of any commercial or financial relationships that could be construed as a potential conflict of interest.
